# Prof. In Kyu Han

**DOI:** 10.5713/ajas.2020.0001M

**Published:** 2019-12-26

**Authors:** Jong Kyu Ha

**Affiliations:** AJAS

The 1st Editor-in-Chief of the Asian-Australasian Journal of Animal Sciences (AJAS), Prof. In Kyu Han, a dedicated teacher and research scientist in animal nutrition, passed away on November 1, 2019 at the age of 85, after a brief illness. Prof. Han was born in Seongjoo, Kyungsang buk-do, Korea on October 12, 1934.

**Figure:**
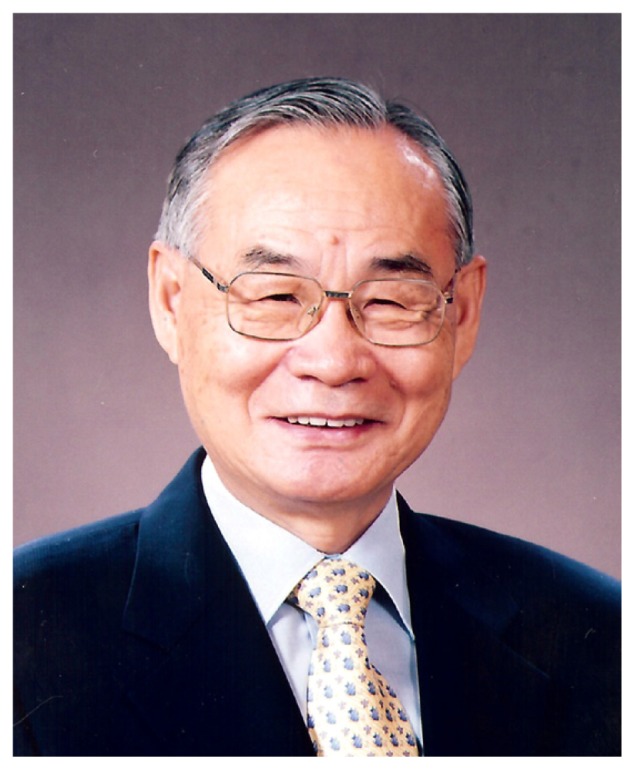


Prof. In K. Han graduated with a degree in Animal Science from Seoul National University in 1956, and an M.Sc. degree from the same institute in 1958, and was awarded a Ph.D. in mono-gastric animal nutrition from Cornell University in 1965. He spent most of his career at the Seoul National University of Korea (1965–2000). As is well known not only in Asia but also worldwide, Prof. Han has been a dedicated teacher, researcher, and administrator for more than 60 years in the area of animal agriculture. He trained more than 170 graduate students, many of whom hold key positions, and contributed to the development of animal science and industry in Korea as well as in the Asian-Australasian Association of Animal Production Societies (AAAP) member countries. As a scientist, Prof. Han published 670 papers in refereed journals, notably in the area of development and utilization of non-conventional animal feeds, amino acids products, and many other ingredients and supplements. Further, he put tremendous effort in the development of Korean feed composition tables and feeding standards for major domestic animal species in Korea, which provided key information and technologies necessary for the modernization of animal agriculture in Korea.

Prof. Han was the type of person with a vision and the ability to play key roles in the engineering of many master plans. The creation and establishment of AAAP in 1980 and AJAS in 1988 are two best examples. Prof. Han with 8 pioneers, including Y. Yamada from Japan and S. Jalaludin from Malaysia, master-minded the establishment of AAAP. He played a key role in the development of the organization until today. He hosted the 3rd AAAP Congress in Seoul, and organized the congress in 1985 as one of the most successful meetings in AAAP history. In addition, Prof. Han strived to establish the official journal of the AAAP ever since its creation, and successfully launched the first issue of AJAS in March 1988. He enriched the journal, both in quantity and quality, from its outset until 2001, when he stepped down as editor-in-chief to take up another important position: President of the Korean Academy of Science and Technology. His international interest was extended to the World Association for Animal Production (WAAP), in which he served as vice president for 5 years, and as president for another 5 years. The 8th World Conference on Animal Production (WCAP), which was organized under his leadership in Seoul in 1998, was and still is the most successful conference in WAAP history.

Within the context of science in Korea, he engineered the establishment of many local scientific societies and their journals: some examples include the Korean Society of Animal Science and Technology, Korean Society of Animal Nutrition and Feed, and Korean Nutrition Society. Prof. Han held a number of important positions in Korea, and is well-known for his achievements in these positions. He served as the dean of the Agricultural University of Seoul National University for 2 years (1989–1991), and was elected as the president of the Korean Academy of Science and Technology for a 3-year term (2001–2004), where he facilitated tremendous achievement for both organizations as well as for the development of sciences including agricultural science in Korea. His affection and interest in fostering human resources for the development of animal science and industry in the AAAP region continued even after his retirement. He established the Hans’ Animal Life Science Foundation with a personal donation of almost one million dollars in 2000. The foundation has provided scholarships to not only Korean students, but also many foreign students. Moreover, his foundation sponsored award programs such as the AAAP Animal Science Award and WAAP International Animal Agriculture Award, and supported international meetings (AAAP and WAAP).

Prof. Han received several awards: the Korean Scientist Award (1971), National Green Ribbon Medal (2000), Korea Academy of Science and Technology Award (2014), Best SNU Award (2015), and AAAP Animal Science Award (2016).

Prof. Han married Mrs. Myung Sook Kim in 1959. He is survived by a son, Changho Han, and three daughters, Miesook Han, Miehie Han, and Miejeong Han. We all share the family members’ sorrow as they go through this most difficult time.

